# A transition-constrained discrete hidden Markov model for automatic sleep staging

**DOI:** 10.1186/1475-925X-11-52

**Published:** 2012-08-21

**Authors:** Shing-Tai Pan, Chih-En Kuo, Jian-Hong Zeng, Sheng-Fu Liang

**Affiliations:** 1 Department of Computer Science and Information Engineering, National University of Kaohsiung, Kaohsiung, 811, Taiwan, R.O.C; 2 Department of Computer Science and Information Engineering, National Cheng Kung University, Tainan, 701, Taiwan, R.O.C

**Keywords:** Sleep Staging, Discrete Hidden Markov Model (DHMM), Electroencephalogram (EEG), Electrooculogram (EOG), Electromyogram (EMG)

## Abstract

**Background:**

Approximately one-third of the human lifespan is spent sleeping. To diagnose sleep problems, all-night polysomnographic (PSG) recordings including electroencephalograms (EEGs), electrooculograms (EOGs) and electromyograms (EMGs), are usually acquired from the patient and scored by a well-trained expert according to Rechtschaffen & Kales (R&K) rules. Visual sleep scoring is a time-consuming and subjective process. Therefore, the development of an automatic sleep scoring method is desirable.

**Method:**

The EEG, EOG and EMG signals from twenty subjects were measured. In addition to selecting sleep characteristics based on the 1968 R&K rules, features utilized in other research were collected. Thirteen features were utilized including temporal and spectrum analyses of the EEG, EOG and EMG signals, and a total of 158 hours of sleep data were recorded. Ten subjects were used to train the Discrete Hidden Markov Model (DHMM), and the remaining ten were tested by the trained DHMM for recognition. Furthermore, the 2-fold cross validation was performed during this experiment.

**Results:**

Overall agreement between the expert and the results presented is 85.29%. With the exception of S1, the sensitivities of each stage were more than 81%. The most accurate stage was SWS (94.9%), and the least-accurately classified stage was S1 (<34%). In the majority of cases, S1 was classified as Wake (21%), S2 (33%) or REM sleep (12%), consistent with previous studies. However, the total time of S1 in the 20 all-night sleep recordings was less than 4%.

**Conclusion:**

The results of the experiments demonstrate that the proposed method significantly enhances the recognition rate when compared with prior studies.

## Introduction

On average, humans spend approximately seven to eight hours a day sleeping. This is equivalent to one-third of the human lifetime, and demonstrates the importance of sleep. At night, an eight hour sleep comprises four or five sleep cycles; each cycle lasts approximately 90 minutes and comprises different stages including light sleep (Stages 1 & 2), deep sleep (Slow Wave Sleep) and rapid eye movement (REM) [1]. The deep sleep stages become shorter as the sleep cycle progresses.

Sleep analysis is not only helpful in diseased conditions but aids several psychophysiological analyses. In human physiology, a good deep sleep (SWS) stage can aid physical recovery; in addition, a good rapid eye movement (REM) stage can improve learning ability and memory. Sleep diseases including insomnia and obstructive sleep apnea, seriously affect a patient’s quality of life. Without restrictive criteria, the prevalence of insomnia symptoms is approximately 33% in the general population. Obstructive sleep apnea affects over 2% of adult women and 4% of adult men [1]. These sleep issues may cause daytime sleepiness, irritability, depression, anxiety or even death.

To diagnose sleep issues, all-night polysomnographic (PSG) recordings including electroencephalograms (EEGs), electrooculograms (EOGs) and electromyograms (EMGs), are usually acquired from patients, and the recordings are scored by a well-trained expert according to the Rechtschaffen & Kales (R&K) rules presented in 1968. According to the R&K rules, each epoch (i.e., 30 s of data) is classified into one of the sleep stages including wakefulness (Wake), non-rapid eye movement (stages 1–4, from light to deep sleep) and rapid eye movement (REM). Recently, stages 3 and 4 were combined and are now known as the slow wave sleep stage (SWS) [2]. Visual sleep scoring is a time-consuming and subjective process. Therefore, automatic sleep staging methods including rule-based methods [3], artificial neural networks (ANN) [4] and hidden Markov models (HMM) [5-7], have been developed. In [5], a GOHMM method with five features calculated from the signals measured by two EEG channels (C3 and C4) and an EMG channel was used for sleep staging. The authors used sleep data from five subjects for training and four subjects for testing. Moreover, in [6] the GOHMM was applied with the signals measured from one EEG channel (C3) for sleep staging. The authors used the sleep data from twenty subjects for training and twenty subjects for testing. In addition, L. G. Doroshenkov et al. [7] explored sleep staging using the HMM method, with the signals measured from two EEG channels (Fpz-Cz and Pz-Oz).

HMM permits analysis of non-stationary multivariate time series by modeling the state transition probabilities and the probability of the observation of a state. During the HMM process, the result of the previous state will influence the state recognition result of the next state. This is similar to the process for sleep staging, which should consider the relationship between the previous sleep stage and the next sleep stage. As it possesses the properties of successive stage transition, the HMM is a promising model for sleep staging.

According to the type of probability distributions used in HMMs, they can be categorized as Continuous Hidden Markov Models (CHMMs) and Discrete Hidden Markov Models (DHMMs). The DHMM provides more stable recognition results and faster training, with a recognition accuracy that is not less than that of the CHMM. Therefore, the DHMM was adopted for sleep staging during this study. The useful features of the sleep signals are selected to train the DHMM. Although HMM-based sleep staging has been explored during previous studies [5-7], the accuracy of these results was poor, probably because sleep stage transition conditions were not included in the HMM modeling process. Indeed, the probability of a sleep stage transition is highly dependent on the current stage; the probability of particular stages appearing after the current stage is low. These transition conditions should be considered during HMM modeling to improve the accuracy of the modeling results, and consequently, the accuracy of sleep staging. Therefore, this report proposed a transition-constrained DHMM based on sleep stage transition conditions. To rule out impossible or infrequent stage transitions, the probability matrices in the proposed transition-constrained DHMM were adjusted to accommodate sleep stage transitions during the training phase to improve the recognition rate. In the future, the constructed HMM model could become a reliable computer-assisted tool for clinical staff to increase the efficiency of sleep scoring.

## Method

**Figure 1:**
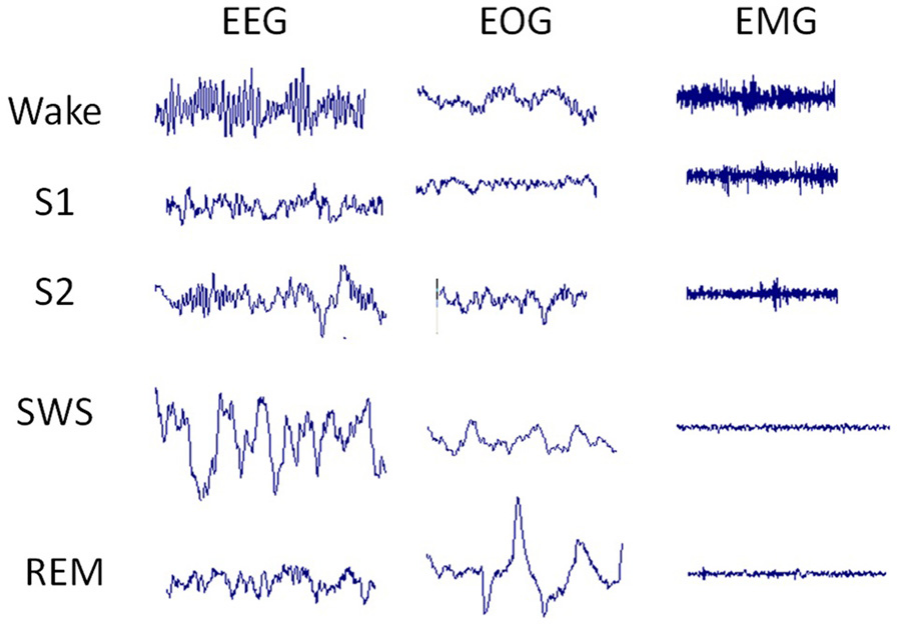
**Typical polygraphic recordings during the wake (WK), stage 1 (S1), stage 2 (S2) light sleep, slow-wave sleep (SWS) associated with deep sleep, and rapid-eye movement (REM) states.** Legend: Each raw shows the electroencephalography (EEG), electrooculography (EOG) and chin electromyography (EMG) data during the corresponding sleep stage.

The main purpose of this section is to provide an understanding of the basic elements of sleep stages. First, polysomnography, which includes electroencephalograms (EEGs), electrooculograms (EOGs) and electromyograms (EMGs), among others, as shown in Figure 1 will be introduced [8]. Second, the classification of sleep stages used in the R&K rules will be introduced, together with an explanation as to why we adopted the new sleep staging classification. Finally, a number of modified sleep stages and the smoothing method for staging results are described.

### Polysomnography

Polysomnography (PSG) is a comprehensive recording of the biophysiological changes that occur during sleep. PSG monitors several body functions during sleep including brain activity (electroencephalogram, EEG), eye movement (electrooculogram, EOG), muscle activity or skeletal muscle activation (electromyogram, EMG), and heart rhythm (electrocardiogram, ECG) [8,9]. During this study, all-night polysomnographic sleep recordings were obtained from 20 healthy subjects (12 males and 8 females) ranging from 19 to 23 years of age (mean = 21.2 ± 1.1 years). These measurements were approved by the internal review board of National Cheng Kung University. The subjects were interviewed concerning their sleep quality and medical history. None of the subjects reported any history of neurological or psychological disorders. The all-night PSGs were recorded in the sleep laboratory at the cognitive institute of National Cheng Kung University. There was no outside interference during data collection, and no medications were used to induce sleep.

The recordings included six EEG channels (F3-A2, F4-A1, C3-A2, C4-A1, P3-A2, and P4-A1, according to the international 10–20 standard system), two EOG channels (positioned 1 cm lateral to the left and right outer canthi), and a chin EMG channel (Siesta 802 PSG, Compumedics, Inc.). The sampling rate was 1 K samples/second with 16-bit resolution. The 20 PSG sleep recordings were visually scored by a sleep specialist using the R&K rules with a 30-s interval (termed the epoch). Figure 1 presents typical polysomnographic recordings corresponding to various sleep stages.

### Visual scoring

The standard of visual sleep staging is based on the R&K rules, which were proposed by Rechtschaffen and Kales in 1968 [10]. According to these rules, stages are scored epoch-by-epoch in 20–30 second intervals. The sleep is divided into six stages: stage wake (Wake), stage 1 (S1), stage 2 (S2), stage 3 (S3), stage 4 (S4) and stage rapid eye movement (REM) [4]. The Rechtschaffen and Kales sleep staging criteria is listed in Table 1.

**Table 1:** **Rechtschaffen and Kales sleep staging criteria**[10]

**Sleep Stage**	**Scoring Criteria**
Wake	When the subject closes their eyes, >50% of the page (epoch) consists of alpha (8–13 Hz) activity or low-voltage, mixed (3–7 Hz) frequency activity.
Stage 1	50% of the page (epoch) consists of related low-voltage mixed (3–7 Hz) activity. Slow rolling eye movements lasting several seconds are often observed in early stage 1.
Stage 2	Appearance of sleep spindles and/or K complexes and <20% of the epoch may contain high-voltage (>75 μV, <2 Hz) activity. Sleep spindles and K complexes must each last >0.5 seconds.
Stage 3	20%-50% of the epoch consists of high-voltage (>75 μV), low-frequency (<2 Hz) activity.
Stage 4	>50% of the epoch consists of high-voltage (>75 μV, <2 Hz) delta activity.
Stage REM	Relatively low-voltage mixed frequency EEG with episodic rapid eye movements and absent or reduced chin EMG activity.

The characteristics of stages S3 and S4 are very similar. Therefore, to facilitate simple and accurate sleep staging, the American Academy of Sleep Medicine (AASM) group combined stages S3 and S4 into the deep sleep, or slow wave sleep (SWS) stage, in 2007 [2]. The term SWS is used to reinforce the physical meaning of this stage. Therefore, during this study the five-stage classification: Wake, S1, S2, SWS, and REM, was utilized.

In addition, if more than half of the EEG or EMG signal epochs were unidentifiable due to amplifier blocking or muscle activity, the epoch was labeled as an arousal stage or a body movement stage. Consequently, a new temporary stage called the movement stage (Mov) was added to the visual scoring if the amplitude of EEG signals was over 200 μV. This temporary movement stage includes arousal and body movement. After the smoothing process at the end of sleep recognition, the movement stage will be replaced by wake if arousal occurs or by other sleep stages if body movement occurs [8].

### Processing for sleep data

The DHMM sleep staging system analyzes data from the central EEG (C3-A2), the difference between the two EOGs, and the chin EMG. After down-sampling the signals to 256 samples/second for lower computational complexity, the EEG and EOG data were filtered with an eighth-order Butterworth band-pass filter with a cutoff frequency of 0.5–30 Hz, and the EMG data were filtered with a 5–100 Hz eighth-order Butterworth band-pass filter. The continuous time signals were segmented with every 30-s epoch.

Before extracting the spectral features, the signal was segmented into non-overlapping intervals of two seconds for a 512-point fast Fourier transformation (FFT) calculation. The spectrums corresponding to the 15 2-s segments were averaged to represent the spectrum for a 30-s epoch. Table 2 lists the 13 features used in this paper [4,9,11-15].

**Table 2:** Features for sleep scoring

**No.**	**Type**	**Feature**	**Source**
1	PS	Total power of 0–30 Hz	EEG
2	PS	Total power of 0–30 Hz	EMG
3	PR	0-4 Hz/0-30 Hz	EEG
4	PR	4-8 Hz/0-30 Hz	EEG
5	PR	8-13 Hz/0-30 Hz	EEG
6	PR	22-30 Hz/0-30 Hz	EEG
7	PR	0-4 Hz/0-30 Hz	EOG
8	SF	Mean frequency of 0–30 Hz	EEG
9	SF	Mean frequency of 0–30 Hz	EMG
10	DR	Alpha ratio	EEG
11	DR	Spindle ratio	EEG
12	DR	SWS ratio	EEG
13	EMG energy	Mean amplitude	EMG

* PS, Power spectrum; PR, Power ratio; SF, Spectral frequency; DR, Duration ratio.

#### Power spectrum (PS)

After the FFT, the power spectrum (dB) was summed among the band 0–30 Hz for the EEG, EOG and EMG, and this was considered the total power. The total powers in the EEG and the EMG were used as features and for the calculation of the power ratio. The total power in the EOG was used for the calculation of the power ratio. It was obtained using the following equation:

(1) $$PS_{\mathrm{total}}=\sum_{f=0}^{30}PS\left(f\right)$$

where *PS*(*f*) is the power of the frequency *f*.

#### Power ratio (PR)

After the FFT, for one frequency band in a 30-sec epoch, which has 15 powers, the mean power of each frequency band was collected. Thereafter, the ratio of each band to the total power 0–30 Hz was calculated and considered a feature. The power ratio is given by the following equation:

(2) $$PR=\frac{\sum_{f=i}^{j}PS\left(f\right)}{\sum_{f=0}^{30}PS\left(f\right)}$$

where *i* and *j* indicate the ranges of the respective spectral power band for the PR features. Table 2 presents the total bands of the power ratio in our features (0–4 Hz, 4–8 Hz, 8–13 Hz, and 22–30 Hz for EEG, and 0–4 Hz for EOG).

#### Spectral frequency (SF)

After FFT, the mean frequency of spectral power (SF) was calculated for the EEG and the EMG. The SF was defined by the equation:

(3) $$SF=\frac{\sum_{f=0}^{30}f\cdot PS\left(f\right)}{\sum_{f=0}^{30}PS\left(f\right)}$$

#### Duration ratio (DR)

##### Alpha ratio

The alpha ratio is the ratio between the alpha windows and the total windows in an epoch. Two eighth-order bandpass Butterworth filters with passbands of 8–13 Hz and 22–30 Hz were designed. In addition to the normally used alpha band of 8–13 Hz, a beta band of 22–30 Hz was added as a feature, as we found that Wake had high power in the 22–30 Hz band. The two filtered signals were combined, and a threshold (0.5) was used to detect it.

##### Spindle ratio

The spindle ratio is the ratio between the spindle windows and the total windows in an epoch. FFT and Butterworth bandpass filtering among the sigma band of 12–15 Hz were used to calculate the spindle ratio. FFT was used to determine if the power of the sigma band (12–15 Hz) was high, and the filtering signal was used to detect any large, sudden amplitude changes.

##### SWS ratio

Similar to the alpha and spindle ratio, the SWS ratio is the ratio between the SWS windows and the total windows in an epoch. We designed a third-order bandpass Butterworth filter with a passband of 0.5-2 Hz. This was predominantly used to separate SWS from the other stages.

#### EMG energy

The mean value of the absolute amplitude of the total data points in an epoch was calculated from the EMG signal and considered a feature. During sleep, particularly during the REM stage, EMG activity decreases compared with the activity while awake. This feature can be used for artifact detection. The EMG energy increases during body movement.

### DHMM for Sleep Staging

This section introduces the proposed strategy for sleep staging by the proposed transition-constrained DHMM. These methods include the method for generating the codebook for the quantization of sleep signal features and the method for modeling the transition-constrained DHMM for sleep recognition.

#### Vector Quantization and Codebook Generation

Each epoch has thirteen features after pre-processing and feature extraction in this study, with the thirteen features being formed as a feature vector. As the feature vectors are real number vectors in real space with thirteen dimensions, vector quantization of these feature vectors is necessary to reduce the computational burden. Therefore, a codebook for the process of feature vector quantization should be created to allow the results of the vector quantization to be a set of observation codes for DHMM implementation and recognition.

To create the codebook, the range of values for each element of the feature vectors in the samples used for training was obtained. The interval between the upper bound and lower bound of the range of each element was divided equally into *m* subintervals, where *m* is the number of groups into which the feature vectors were classified. Thereafter, *m* vectors were generated to form the initial codebook. Initially, the value of each element of the *m* vectors was randomly generated with a value in the corresponding subinterval. To train the codebook, we calculated the distance *d*_*k*_ between the feature vector *v*_*f*_ = [*v*_*f*0_*v*_*f*1_…*v*_*fn*_]^*T*^ and the *kth* vector *V*_*ck*_ = [*V*_*ck*0_*V*_*ck*1_…*V*_*ckn*_]^*T*^ in the codebook as follows:

(4) $$d_{k}\left(v_{f}\right)=\sqrt{\sum_{i=0}^{n}{\left(v_{\mathrm{fi}}-V_{\mathrm{cki}}\right)}^{2}}$$

After obtaining all values of *d*_*k*_ through the codebook, *v*_*f*_ was classified in the group with the smallest *d*_*k*_. According to the updated feature vectors of each group, a new center vector (a row vector in the codebook) was obtained:

(5) $${\hat{V}}_{k}=\frac{1}{N_{k}}\times \sum_{n=1}^{N_{k}}v_{\mathrm{fn}}^{k}$$

where V_*k*_ is the new center vector (a new row vector in codebook) of the *kth* group, *v*_*fi*_^*k*^ is the *ith* feature vector in the *kth* group, and *N*_*k*_ is the number of feature vectors in the *kth* group. These two steps were repeated until the codebook converged, completing the training process of the codebook.

#### DHMM and Sleep Stage

**Figure 2:**
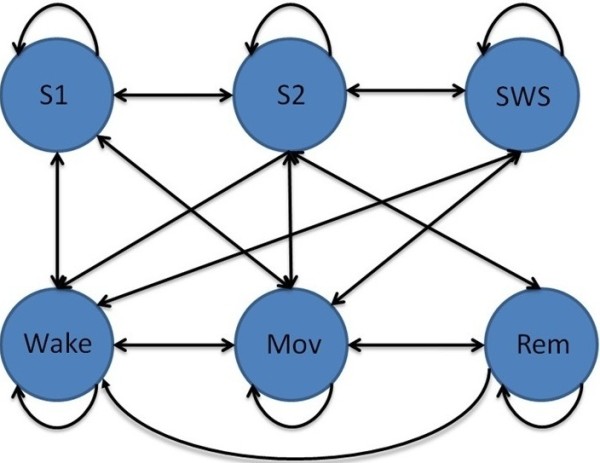
Allowable sleep stage transitions in healthy subjects.

During sleep staging, the current stage usually has a significant relationship with the next stage. That is, the next stage will transit based on the current stage. For example, if the current stage is Wake, the next stage may be Wake or S1. Hence, the probability of a specific sleep stage transition is highly dependent on the current stage. The probability of a certain stage appearing after the current stage is limited. For example, the probability of Wake appearing after SWS is low [8]. Based on the expert manual staging results, the sleep stage transition rules were obtained and are depicted in Figure 2; all of the allowable transitions are indicated by arrows.

While the states of the DHMM are unobservable, the outputs in each state are observable. Each state has a probability distribution over the possible output tokens. Therefore, the sequence of output tokens generated by the DHMM provides information concerning the sequence of hidden states. To apply the DHMM to sleep staging, the features introduced in Sec. II were defined as the output tokens (observation), while the sleep stages were defined as the hidden states of the DHMM. The parameters for the DHMM [16] were defined as follows:

λ:, DHMM model, λ = (A, B, π); A:, A = [aij], aij is the probability of state xi transferring to state xj; B:, B = [bj(k)], bj(k) is the probability of the kth observation, which is observed from state xj, i.e., bj(k) = P(ot = vk|qt = xj); π:, π = [πi], πi is the probability of the case where the initial state is xi; X:, the state vectors of the DHMM; V:, the observation event vector of the DHMM; O:, the observation results of the DHMM; Q:, the resulting states of the DHMM; Eij, the event of the transition from state xi to state xj; Ei·, the event of the transition from state xi to leave; E·j, the event of the transition from other states to state xj; Ehi, the event of state xi appears at the initial state; n(Eij), the number of the transition from state xi to state xj; n(Ei·), the number of the transition from state xi to other states; n(E·j), the number of the transition from other states to state xj; n(E·j, o = vk), the number of the transition from other states to state xjwith observation code vk; n(Ehi), the number of the event of state xi appears at the initial state.

(6) $$a_{\mathrm{ij}}=P\left(q_{t}=x_{j}|q_{t-1}=x_{i}\right)$$

λ:, DHMM model, λ = (A, B, π); A:, A = [aij], aij is the probability of state xi transferring to state xj; B:, B = [bj(k)], bj(k) is the probability of the kth observation, which is observed from state xj, i.e., bj(k) = P(ot = vk|qt = xj); π:, π = [πi], πi is the probability of the case where the initial state is xi; X:, the state vectors of the DHMM; V:, the observation event vector of the DHMM; O:, the observation results of the DHMM; Q:, the resulting states of the DHMM; Eij, the event of the transition from state xi to state xj; Ei·, the event of the transition from state xi to leave; E·j, the event of the transition from other states to state xj; Ehi, the event of state xi appears at the initial state; n(Eij), the number of the transition from state xi to state xj; n(Ei·), the number of the transition from state xi to other states; n(E·j), the number of the transition from other states to state xj; n(E·j, o = vk), the number of the transition from other states to state xjwith observation code vk; n(Ehi), the number of the event of state xi appears at the initial state.

(7) $$\pi_{i}=P\left(q_{1}=x_{i}\right)$$

λ:, DHMM model, λ = (A, B, π); A:, A = [aij], aij is the probability of state xi transferring to state xj; B:, B = [bj(k)], bj(k) is the probability of the kth observation, which is observed from state xj, i.e., bj(k) = P(ot = vk|qt = xj); π:, π = [πi], πi is the probability of the case where the initial state is xi; X:, the state vectors of the DHMM; V:, the observation event vector of the DHMM; O:, the observation results of the DHMM; Q:, the resulting states of the DHMM; Eij, the event of the transition from state xi to state xj; Ei·, the event of the transition from state xi to leave; E·j, the event of the transition from other states to state xj; Ehi, the event of state xi appears at the initial state; n(Eij), the number of the transition from state xi to state xj; n(Ei·), the number of the transition from state xi to other states; n(E·j), the number of the transition from other states to state xj; n(E·j, o = vk), the number of the transition from other states to state xjwith observation code vk; n(Ehi), the number of the event of state xi appears at the initial state.

(8) $$X=\left(x_{1},x_{2},\cdot \cdot \cdot ,x_{N}\right)$$

λ:, DHMM model, λ = (A, B, π); A:, A = [aij], aij is the probability of state xi transferring to state xj; B:, B = [bj(k)], bj(k) is the probability of the kth observation, which is observed from state xj, i.e., bj(k) = P(ot = vk|qt = xj); π:, π = [πi], πi is the probability of the case where the initial state is xi; X:, the state vectors of the DHMM; V:, the observation event vector of the DHMM; O:, the observation results of the DHMM; Q:, the resulting states of the DHMM; Eij, the event of the transition from state xi to state xj; Ei·, the event of the transition from state xi to leave; E·j, the event of the transition from other states to state xj; Ehi, the event of state xi appears at the initial state; n(Eij), the number of the transition from state xi to state xj; n(Ei·), the number of the transition from state xi to other states; n(E·j), the number of the transition from other states to state xj; n(E·j, o = vk), the number of the transition from other states to state xjwith observation code vk; n(Ehi), the number of the event of state xi appears at the initial state.

(9) $$V=\left(v_{1},v_{2},\cdot \cdot \cdot ,v_{M}\right)$$

λ:, DHMM model, λ = (A, B, π); A:, A = [aij], aij is the probability of state xi transferring to state xj; B:, B = [bj(k)], bj(k) is the probability of the kth observation, which is observed from state xj, i.e., bj(k) = P(ot = vk|qt = xj); π:, π = [πi], πi is the probability of the case where the initial state is xi; X:, the state vectors of the DHMM; V:, the observation event vector of the DHMM; O:, the observation results of the DHMM; Q:, the resulting states of the DHMM; Eij, the event of the transition from state xi to state xj; Ei·, the event of the transition from state xi to leave; E·j, the event of the transition from other states to state xj; Ehi, the event of state xi appears at the initial state; n(Eij), the number of the transition from state xi to state xj; n(Ei·), the number of the transition from state xi to other states; n(E·j), the number of the transition from other states to state xj; n(E·j, o = vk), the number of the transition from other states to state xjwith observation code vk; n(Ehi), the number of the event of state xi appears at the initial state.

(10) $$O=o_{1},o_{2},\cdot \cdot \cdot ,o_{T}$$

λ:, DHMM model, λ = (A, B, π); A:, A = [aij], aij is the probability of state xi transferring to state xj; B:, B = [bj(k)], bj(k) is the probability of the kth observation, which is observed from state xj, i.e., bj(k) = P(ot = vk|qt = xj); π:, π = [πi], πi is the probability of the case where the initial state is xi; X:, the state vectors of the DHMM; V:, the observation event vector of the DHMM; O:, the observation results of the DHMM; Q:, the resulting states of the DHMM; Eij, the event of the transition from state xi to state xj; Ei·, the event of the transition from state xi to leave; E·j, the event of the transition from other states to state xj; Ehi, the event of state xi appears at the initial state; n(Eij), the number of the transition from state xi to state xj; n(Ei·), the number of the transition from state xi to other states; n(E·j), the number of the transition from other states to state xj; n(E·j, o = vk), the number of the transition from other states to state xjwith observation code vk; n(Ehi), the number of the event of state xi appears at the initial state.

(11) $$Q=q_{1},q_{2},\cdot \cdot \cdot ,q_{T}$$

Note that, for the application of the DHMM to sleep staging, the states *x*_*i*_, *i* = 1, 2, …, 6 in the DHMM correspond to the sleep stages Wake, S1, S2, SWS, REM, and Mov, respectively.

#### Training the transition-constrained DHMM

This subsection introduces training of the transition-constrained DHMM based on the allowable sleep stage transitions in Figure 2. First, the probability of the observations according to the DHMM model *λ* = (*A*, *B*, *π*) was calculated using the following equation (6) [17]:

(12) $$P\left(O|\lambda \right)=\sum_{Q}P\left(O|Q,\lambda \right)P\left(Q|\lambda \right)=\sum_{q_{1}\ldots q_{T}}\pi_{q_{1}}b_{q_{1}}\left(o_{1}\right)\cdot a_{q_{1}q_{2}}b_{q_{2}}\left(o_{2}\right)\cdot \cdot \cdot a_{q_{T-1}q_{T}}b_{q_{T}}\left(o_{T}\right)\text{.}$$

This equation enables an evaluation of the probability of the observations *O* based on the DHMM model *λ* = (*A*, *B*, *π*). However, the amount of time needed to evaluate *P*(*O*|*λ*) directly would be exponential to the observation number *T*. For this reason, a Forward Algorithm [16] was applied to reduce the computation time for equation (6) and is described as follows.

##### The forward algorithm

The Forward Algorithm can be described by three steps: initialization, recursion and termination. The details are listed below and are depicted in Figure 2.

Initialization:

(13) $$\alpha_{1}\left(i\right)\equiv \pi_{i}b_{i}\left(o_{1}\right),1\leq i\leq N$$

Recursion:

(14) $$\alpha_{t+1}\left(j\right)=\left[\sum_{i=1}^{N}\alpha_{t}\left(i\right)a_{\mathrm{ij}}\right]b_{j}\left(o_{t+1}\right),1\leq t\leq T-1,1\leq j\leq N$$

Termination:

(15) $$P\left(O|\lambda \right)=\sum_{i=1}^{N}\alpha_{T}\left(i\right)$$

The Forward Algorithm reduces the complexity of the calculations from 2*TN*^*T*^ to *N*^2^*T*[16].

To train the DHMM model parameters *λ* = (*A*, *B*, *π*) based on the sleep data, some notations were defined for convenience as follows:

λ:, DHMM model, λ = (A, B, π); A:, A = [aij], aij is the probability of state xi transferring to state xj; B:, B = [bj(k)], bj(k) is the probability of the kth observation, which is observed from state xj, i.e., bj(k) = P(ot = vk|qt = xj); π:, π = [πi], πi is the probability of the case where the initial state is xi; X:, the state vectors of the DHMM; V:, the observation event vector of the DHMM; O:, the observation results of the DHMM; Q:, the resulting states of the DHMM; Eij, the event of the transition from state xi to state xj; Ei·, the event of the transition from state xi to leave; E·j, the event of the transition from other states to state xj; Ehi, the event of state xi appears at the initial state; n(Eij), the number of the transition from state xi to state xj; n(Ei·), the number of the transition from state xi to other states; n(E·j), the number of the transition from other states to state xj; n(E·j, o = vk), the number of the transition from other states to state xjwith observation code vk; n(Ehi), the number of the event of state xi appears at the initial state.

**Figure 3:**
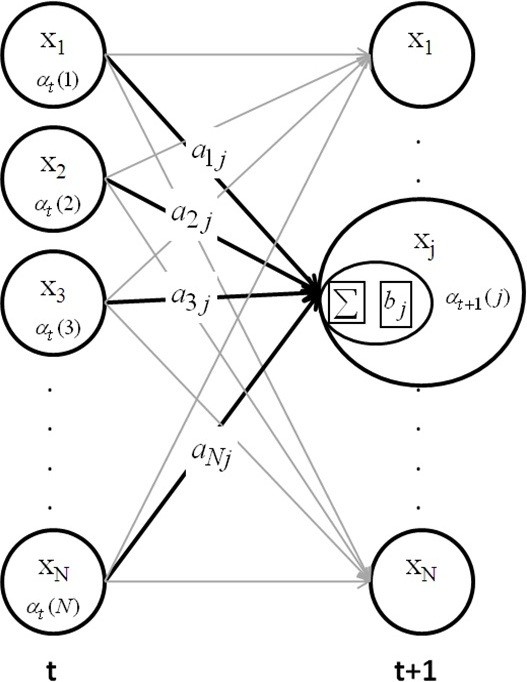
Illustration of the Forward Algorithm.

To train *A*, *B*, and*π* of the DHMM, the hidden states for each observation were estimated with the initial *A*, *B*, and*π*. Thereafter, the values *n*(*E*_*ij*_), *n*(*E*_*i*·_), *n*(*E*_·*j*_), *n*(*E*_·*j*_, *o* = *v*_*k*_), and *n*(*E*_*hi*_) were computed for all training data. Subsequently, the elements in matrices *A*, *B*, and*π* were updated as follows,

(16) $$\bar{a_{\mathrm{ij}}}=\frac{n\left(E_{\mathrm{ij}}\right)}{n\left(E_{i\cdot }\right)}$$

(17) $$\bar{b_{j}}\left(k\right)=\frac{n\left(E_{\cdot j},o=v_{k}\right)}{n\left(E_{\cdot j}\right)}$$

(18) $$\bar{\pi_{i}}=\frac{n\left(E_{\mathrm{hi}}\right)}{n_{\mathrm{TD}}}\text{,}$$

where *n*_*TD*_ is the number of training data. The transition matrix *A* stores the probability of state *x*_*j*_ following state *x*_*i*_. In the proposed transition-constrained DHMM, to rule out impossible sleep stage transitions, the *a*_*ij*_ corresponding to the impossible transition was set to zero according to the sleep stage transition diagram presented in Figure 2. For example, the situation of SWS following Wake is impossible, and *a*_14_ is set to zero. The above procedures were repeated until the matrices *A*, *B*, and*π* converged. Figure 3 illustrates the training process for the DHMM sleep model.

#### States guess from DHMM

The states hidden behind the DHMM can be estimated according to the measured observation of each state by the DHMM. This is the most important process in applying the DHMM to sleep staging. In this paper, the Viterbi Algorithm [16] was used to calculate the hidden states in the DHMM:

##### *The viterbi algorithm*

Initialization:

(19) $$\delta_{1}\left(i\right)\equiv \pi_{i}b_{i}\left(o_{1}\right),1\leq i\leq N$$

Recursion:

(20) $$\delta_{t}\left(j\right)=\max_{1\leq i\leq N}\left[\delta_{t-1}\left(i\right)a_{\mathrm{ij}}\right]b_{j}\left(o_{t}\right),2\leq t\leq T,1\leq j\leq N$$

(21) $$\psi_{t}\left(j\right)=\arg\max_{1\leq i\leq N}\left[\delta_{t-1}\left(i\right)a_{\mathrm{ij}}\right],2\leq t\leq T,1\leq j\leq N$$

Termination:

(22) $$P^{*}=\max_{1\leq i\leq N}\left[\delta_{T}\left(i\right)\right]$$

(23) $$q_{T}^{*}=\arg\max_{1\leq i\leq N}\left[\delta_{T}\left(i\right)\right]\text{,}$$

where *q*_T_^*^ is the estimated state in time *T*.

Optimal state sequence backtracking for estimated states *q*_t_^*^:

(24) $$q_{t}^{*}=\psi_{t+1}\left(q_{t+1}^{*}\right),t=T-1,T-2,\ldots ,1.$$

**Figure 4:**
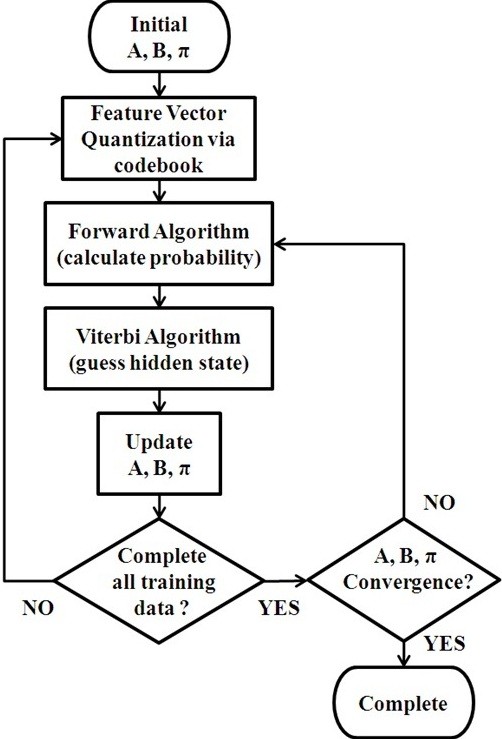
The DHMM sleep model training process.

In the Viterbi Algorithm, *δ*_*t*_(*j*) records the maximum probability transition from the previous state *x*_*i*_ to the present state *x*_*j*_, and *ψ*_*t*_(*j*) records the state with the estimated highest possibility from the previous state. The Viterbi Algorithm used for calculating the state of the DHMM from equations (13)-(18) is illustrated in Figure 4 for clarification.

#### Proposed strategy for sleep staging

**Figure 5:**
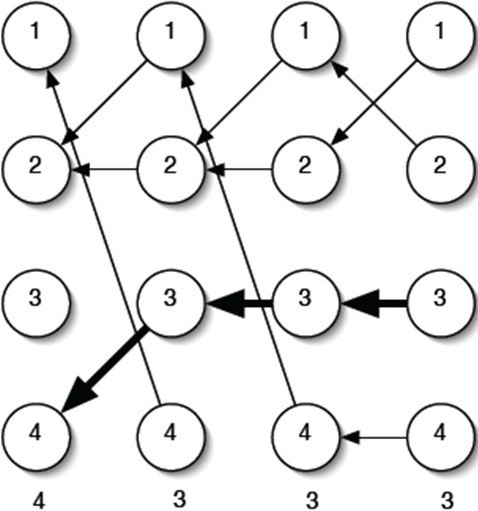
**Illustration of the backtracking step of the Viterbi Algorithm**[16].

In this section, the strategy for sleep staging is described. In the DHMM recognition process, the transition-constrained DHMM model should be trained and then used for sleep staging. As the transitions between hidden states of the DHMM are similar to those between the sleep stages, the sleep stages were assigned to be the hidden stages of the DHMM (see Figure 5). Consequently, for the DHMM *λ* = (*A*, *B*, *π*) applied to sleep staging, the matrix *A* indicates the probability of transition from one sleep stage to another, the matrix *B* indicates the probability of the observation from the sleep stage to which the feature vector belongs, and the matrix *π* indicates the probability that a sleep stage occurs during the initial stage in a sleep sample.

**Figure 6:**
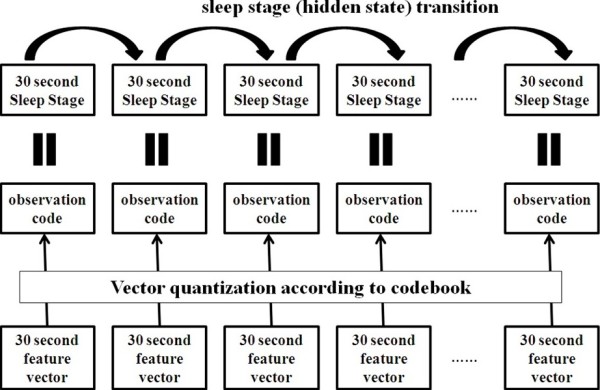
Structure of a DHMM for sleep staging.

The features of each epoch via vector quantization become the observations and were used to train the DHMM or estimate the sleep stage by the DHMM. Each observation code, which is the feature vector calculated from a 30-second sample of a sleep stage, was used to estimate the sleep stage. Therefore, the total sleep stages for a sleep sample could be estimated by testing a sequence of observation codes through the DHMM. Figure 6 presents the structure of a DHMM used for sleep staging.

Based on the discussion above, the sleep staging procedure is summarized as follows:

Step 1. Train codebook by *K*-mean method using the sleep signal samples for training.

Step 2. Generate DHMM model by training (*A*, *B*, *π*) under the constraints of the sleep stage transitions given in Figure 2.

Step 3. Create a quantized observation code from an epoch’s feature vector to be quantized using the codebook in Step 1.

Step 4. Perform the Forward Algorithm using (*A*, *B*, *π*) in the DHMM. Recognize the sleep stage with the highest probability at the end of the sleep stage sample.

Step 5. Backtrack using the Viterbi Algorithm to deduce the transference of all sleep stages.

### Statistics

To evaluate the performance of the proposed sleep staging method, 10 all-night PSG recordings from 10 subjects were used for testing. The Cohen’s kappa coefficient [18] was calculated to assess the robustness of our system. Cohen’s kappa coefficient (*K*) is a statistical measure of inter-rater agreement among two or more raters. The Cohen's kappa *K* is defined by equation (19):

(25) $$K=\left(P_{o}-P_{e}\right)/\left(1-P_{e}\right)$$

where *P*_*o*_ is the relative observed agreement among raters or the total agreement probability, and *P*_*e*_ is the hypothetical probability of chance agreement. This is thought to be a more robust measure than simple percent agreement calculations, as *κ* considers agreements that occur by chance. The interpretation of kappa coefficients by Landis and Koch [19] is as follows: values of less than 0.00 indicate poor agreement; 0.00 to 0.20 indicate slight agreement; 0.21 to 0.40 indicate fair agreement; 0.41 to 0.60 indicate moderate agreement; 0.61 to 0.80 indicate substantial agreement; and greater than 0.80 indicate excellent agreement. The average kappa (0.73) of our system demonstrated substantial reliability.

## Experiment

This section will reveal the experimental results based on the strategy described in the previous section. The EEG, EOG and EMG signals of twenty subjects between the ages of nineteen and twenty-seven years were measured. These data were then used for the sleep staging experiment. The specs used during the experiments for the DHMM are presented in Table 3. The experimental results are described and discussed in the following subsections.

**Table 3:** Summary of the specs of the DHMM in the experiments

**Items**	**Specs of the DHMM**
Time duration for one epoch	**30 seconds**
Epoch number for a subject	**900**
Number of training data	**10**
Number of testing data	**10**
Size of codebook	**30/40/50/60** (four different codebook sizes were used)
Hidden state	**5** (equal to the five sleep stages)
Observation code	**30/40/50/60** (equal to the codebook size)
*λ* = (*A*, *B*, *π*)	
*A*	**matrix dimensions equal to Hidden state**× **Hidden state**
*B*	**matrix dimensions equal to Observation code**× **Hidden state**
*π*	**matrix dimensions equal to Hidden state**× **1**

### Experiment results

During the experiment, we used the signals measured from single EEG channel (C3-A2), EMG and EOG to generate the thirteen features. A total of 158 hours of sleep data were recorded and then used to train ten subjects and test ten subjects.

The performance of different codebook sizes and their respective recognition rates were compared using the DHMM. The results are listed in Table 4, where the mean of the recognition results is defined as the number of correctly recognized epochs over the number of the total epochs in a sample subject. The mean value of the total sleep stage recognition rate (*M*_*SSRR*_) is determined by equation (20):

(26) MSSRR=∑i=15CiET

where *C*_*i*_ is the number of correct recognitions for the *ith* stage and *E*_*T*_ is the total epochs.

**Table 4:** Recognition rates and kappa coefficient for different sizes of codebook by DHMM using ten testing subjects

**codebook = 30**	**DHMM (*M***_***SSRR***_**: 83.39% and*K* = 0.69)**					
**Manual scoring**		Wake	S1	S2	SWS	REM
	Wake (%)	**81.11**	8.87	7	0.09	2.93
	S1 (%)	24.38	**26.6**	31.08	3.96	13.97
	S2 (%)	4.77	1.24	**77.03**	11.58	5.39
	SWS (%)	0.55	0	3.54	**95.92**	0
	REM (%)	0	1.72	2.94	0.49	**94.84**
**codebook = 40**	**DHMM (*M***_***SSRR***_**: 84.36%** ***K* = 0.71)**					
**Manual scoring**	Wake (%)	**86.82**	4.29	6.63	0.09	2.17
	S1 (%)	29.17	**30.31**	28.59	0.20	11.72
	S2 (%)	2.94	1.14	**82.41**	10.95	4.35
	SWS (%)	0.31	0	4.55	**95.14**	0
	REM (%)	0	5.85	5.33	0.11	**88.7**
**codebook = 50**	**DHMM (*M***_***SSRR***_**: 85.29%** ***K* = 0.73)**					
**Manual scoring**	Wake (%)	**88.81**	2.64	6.29	0.09	2.17
	S1 (%)	21.44	**33.62**	33.19	0.21	11.54
	S2 (%)	2.16	1.08	**81.58**	11.46	3.72
	SWS (%)	0.23	0	4.85	**94.92**	0
	REM (%)	0	3.47	5.05	1.34	**90.14**
**codebook = 60**	**DHMM (*M***_***SSRR***_**: 85.12%** ***K* = 0.69)**					
**Manual scoring**	Wake (%)	**83.68**	7.57	5.82	0.08	2.84
	S1 (%)	26.41	**26.71**	33.2	0	13.67
	S2 (%)	2.66	1.66	**82.3**	9.82	3.56
	SWS (%)	0	0	7.24	**92.76**	0
	REM (%)	1.23	2.3	6.07	0	**90.4**

According to the results in Table 4, we can conclude that a larger codebook size contributes to better recognition rates (i.e., codebook = 50 or 60). However, when the size of the codebook reaches 60, the recognition rate no longer increases, and the kappa coefficient decreases. Moreover, the training time increases greatly when the size of the codebook reaches 60. Thus, a codebook size of 50 was chosen as the optimal number for the experiments.

With the exception of S1, the sensitivities of the stages were higher than 81%. The most accurately classified stage was SWS (94.9%), and the least accurately classified stage was S1 (<34%). In the majority of cases, S1 was classified as Wake (21%), S2 (33%) or REM sleep (12%), consistent with previous studies [5-7]. However, the total time of S1 in the 20 all-night sleep recordings was less than 4%.

Moreover, Table 5 presents the subject-by-subject percentage agreements and Cohen’s kappa coefficients of the manual scoring versus automatic scoring. The overall agreement of each subject ranged from 77.09% to 92.62%, and the average sensitivity was 85.29% (S.D. = 5.5). These results demonstrate that the proposed sleep scoring method can achieve stable performance across subjects. The average kappa value was *κ* = 0.73 (S.D. = 0.05), and the individual kappas ranged from 0.64 to 0.78 for the PSGs of 10 subjects. The results represented a substantial agreement between our method and the scoring of the expert.

**Table 5:** Subject-by-subject agreement percentages and Cohen’s kappa coefficients

**Subject No.**	**Wake (%)**	**S1 (%)**	**S2 (%)**	**SWS (%)**	**REM (%)**	**Overall (%)**	**Kappa**
1	100	38.43	86.89	93.97	85.19	85.22	0.77
2	95.31	67.35	83.73	87.5	78.76	83.71	0.78
3	100	24.66	89.42	100	90.44	90.41	0.77
4	93.33	8.53	69.53	100	83.91	78.95	0.64
5	100	31.33	67.77	100	87.82	77.09	0.72
6	75	28.77	93.78	96.84	87.26	92.14	0.71
7	100	22	90.84	100	97.33	92.62	0.78
8	100	9.11	76.97	78.91	94.96	80.26	0.65
9	84.48	48	71.59	97.51	100	84.11	0.76
10	40	58	85.25	94.44	95.76	88.35	0.69
Mean (std)	88.81 (19.1)	33.62 (19.1)	81.58 (9.4)	94.92 (6.9)	90.14 (6.8)	85.29 (5.5)	0.73 (0.05)

S.D., standard deviation.

The 2-fold cross validation was performed during our experiment. The data set was divided into two subsets, each containing ten subjects. Each time one of the two subsets is used as the test set and the other as a training set. This evaluation process was repeated five times with random shuffling of the training–testing datasets. The average overall agreement was 84.68% (S.D. 1.61%). This result demonstrated the robustness of our method.

### Comparison with the existing literature

To compare the results of this paper with existing research concerning HMM-based sleep staging, previous experimental results are listed in Table 6. Each of these papers was published prior to 2007. Therefore, the six-stage (Wake, S1, S2, S3, S4, and REM) classification method was used. To compare these results with our experiment, the recognition rates of S3 and S4 in the existing literature were summed in the SWS stage. In addition, the kappa coefficient of the existing research was calculated and is presented in Table 6.

**Table 6:** Recognition rate and kappa coefficient in other research

**(a) GOHMM**[5]**(**kappa = 0.36**)**						
**Manual scoring**		Wake	S1	S2	SWS	REM
	Wake (%)	**86**	11	0	0	3
	S1 (%)	52	**22**	6	7	13
	S2 (%)	13	12	**14**	24	37
	SWS (%)	1	0	4	**95**	0
	REM (%)	32	16	13	13	**26**
**(b) GOHMM**[6]**(**kappa = 0.50**)**						
**Manual scoring**	Wake (%)	**79**	10	4	0	7
	S1 (%)	21	**24**	19	5	31
	S2 (%)	3	8	**36**	24	29
	SWS (%)	0	0	6.5	**93.5**	0
	REM (%)	14	13	4	1	**68**
**(c) HMM**[7]**(**kappa = 0.52**)**						
**Manual scoring**	Wake (%)	**51.04**	47.30	0.41	0	1.24
	S1 (%)	0	**4.84**	48.39	0	46.77
	S2 (%)	0	0	**68.62**	29.34	2.04
	SWS (%)	0	1.09	0	**98.91**	0
	REM (%)	0	0.94	4.72	8.02	**86.32**

Table 6 (a) includes the results from the Austrian Research Institute for Artificial Intelligence, 2002 [5], where the authors used a GOHMM method with five features calculated from the signals measured by two EEG channels (C3 and C4) and EMG recorded during sleep. The authors used sleep data from five subjects for training and four subjects for testing. Table 6 (b) presents the results from [6], in which the GOHMM was used with the signals measured from a single EEG channel (C3) during sleep. In [6], sleep data from twenty subjects were used for training and twenty subjects for testing. The results in Table 6 (c) are from the study by L. G. Doroshenkov, 2007 [7], in which the HMM method was used with the signals measured from two EEG channels (Fpz-Cz and Pz-Oz). The sleep data for these experiments are from the international database PhysioNet [20]. The kappa coefficient of our method is higher than those of the aforementioned studies.

**Figure 7:**
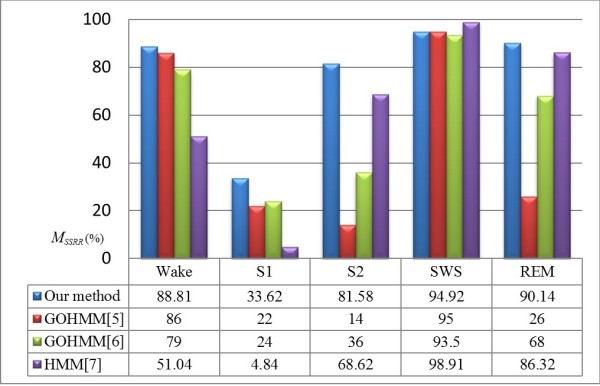
Comparison of the recognition results for each sleep stage obtained in this study with those of existing studies.

Figure 7 presents the recognition rates of the five sleep stages in this paper and the existing research concerning HMM-based sleep staging. The average accuracy of our method was as follows: Wake, 88.81%; S1, 33.62%; S2, 81.58%; SWS, 94.92%; and REM, 90.14%. The previous three methods average accuracy were as follows: Wake, 86%; S1, 22%; S2, 14%; SWS, 95%; and REM, 26% in [5]; in [6], Wake, 51.04%; S1, 4.84%; S2, 68.62%; SWS, 98.91%; and REM, 96.32%; and in [7], Wake, 79%; S1, 24%; S2, 36%; SWS, 93.5%; and REM, 68%. The correct recognition rates of our method are better than those of the other methods for the five sleep stage, with the exception of SWS, although the differences between the methods for this stage were minimal (≤4%). Moreover, the epoch number of S2 is largest, accounting for approximately 40-45% of all night sleep in normal subjects [17], and the correct recognition rate of S2 by our proposed method is better than that of the other methods by between 13% and 67%.

## Conclusions

In this paper we proposed a new strategy based on DHMMs, a transition-constrained DHMM, for sleep staging. The establishment of characteristic features is very important for sleep staging and we chose thirteen features from EEGs, EOGs and EMGs. These features are helpful in distinguishing sleep stages. Although the number of features used in this study is greater than that of previous studies, the recognition time for sleep stages is not longer, and the recognition rate is better.

Although several studies based on HMMs have demonstrated high accuracy in SWS, the sensitivity of S1 was only approximately 20% [7]. S1 is easily mistaken as one of the other stages, with the exception of SWS, and the number of S1 epochs is significantly lower than that of the other stages. Therefore, it is difficult to train a model with a high sensitivity for S1. In our approach, the sensitivity of S1 was approximately 33%. Compared with other research referred to, our S1 result is the most sensitive.

A further advantage of our method is that the recognition rates of each stage are very balanced. The average kappa (0.73) of our system exhibited substantial reliability and high robustness. From a clinical perspective, some stages have high error rates and cannot be used in clinical applications, even if a stage has high sensitivity. Compared with existing results, our method has the highest kappa coefficient and good home healthcare applicability.

Moreover, a smoothing process was not required; this is an advantage of the transition-constrained DHMM, which already considers the relationship between sleep stages in transitions. Sleep staging has periodicity and continuity from light to deep. However, general classifiers such as the neural network, fuzzy system and rule-based methods, do not consider temporal contextual information. Therefore, some epochs may be staged with apparent error, and we should modify these erroneous judgments according to the temporal contextual information and R&K rules. We applied smoothing rules mentioned in previous studies [2,15] to increase the accuracy of our proposed method. However, the smoothing process did not significantly improve the recognition rate. Therefore, we can reduce the time and computing cost associated with the smoothing process using our proposed method.

To implement the proposed transition-constrained DHMM, the parameters in the DHMM corresponding to the impossible transition will be set to zero in the training phase to prevent impossible sleep stage transitions. This improves the recognition rate of the HMM-based method. The results demonstrate that the recognition rates in our proposed method are greatly enhanced when compared with existing research. In the future these results, which were obtained using young, healthy individuals, should be extended to older healthy individuals and patients. This method can be applied clinically to reduce the scoring time. Moreover, we will combine this algorithm with hardware to develop a portable polysomnography system for home healthcare [21]. DHMM models consider the continuity of human sleep based on probabilistic principles in model construction [6]. In addition to automatic sleep scoring applications, future work will include evaluating the continuity of sleep scoring resulting from the DHMM model, cross experts and the conventional smoothing to enhance the agreement between experts and the machine.

## Competing interests

The authors declare that they have no competing interests.

## Authors’ contributions

All authors conceived the study, evaluated the data, and performed data analyses. CEK and JHZ recruited subjects, managed data acquisition, and drafted the manuscript. STP and SFL supervised the study. All authors read, reviewed and approved the final manuscript.
